# Mediators and moderators in the relationship between maternal childhood adversity and children's emotional and behavioural development: a systematic review and meta-analysis

**DOI:** 10.1017/S0033291722001775

**Published:** 2022-07

**Authors:** Xuemei Ma, Alessandra Biaggi, Chiara Sacchi, Andrew J. Lawrence, Pei-Jung Chen, Rebecca Pollard, Maryam Matter, Nuria Mackes, Katie Hazelgrove, Craig Morgan, Seeromanie Harding, Alessandra Simonelli, Gunter Schumann, Carmine M. Pariante, Mitul Mehta, Giovanni Montana, Ana Rodriguez-Mateos, Chiara Nosarti, Paola Dazzan

**Affiliations:** 1 Department of Psychological Medicine, Institute of Psychiatry, Psychology, and Neuroscience, King's College London, London, UK; 2 Department of Developmental Psychology and Socialisation, University of Padova, Padua, Italy; 3 National Institute for Health Research (NIHR) Mental Health Biomedical Research Centre at South London and Maudsley NHS Foundation Trust and King's College London, London, UK; 4 Department of Health Service & Population Research, Institute of Psychiatry, Psychology, and Neuroscience, King's College London, London, UK; 5 Division of Diabetes and Nutritional Sciences, King's College London, London, UK; 6 Biological Psychiatry, Institute of Psychiatry, Psychology and Neuroscience, King's College London, London, UK; 7 Department of Neuroimaging & Psychopharmacology, Centre of Neuroimaging Sciences, King's College London, London, UK; 8 Department of Data Science, University of Warwick, Coventry, UK; 9 Department of Nutritional Sciences, School of Life Course and Population Sciences, Faculty of Life Sciences and Medicine, King's College London, London, UK; 10 Department of Child and Adolescent Psychiatry, Institute of Psychiatry, Psychology, and Neuroscience, King's College London, London, UK; 11 Centre for the Developing Brain, Department of Perinatal Imaging & Health, School of Biomedical Engineering & Imaging Sciences, King's College London, London, UK

**Keywords:** Child emotional and behavioural development, ecological framework, maternal childhood adversity, mediator, moderator

## Abstract

Maternal experiences of childhood adversity can increase the risk of emotional and behavioural problems in their children. This systematic review and meta-analysis provide the first narrative and quantitative synthesis of the mediators and moderators involved in the link between maternal childhood adversity and children's emotional and behavioural development. We searched EMBASE, PsycINFO, Medline, Cochrane Library, grey literature and reference lists. Studies published up to February 2021 were included if they explored mediators or moderators between maternal childhood adversity and their children's emotional and behavioural development. Data were synthesised narratively and quantitatively by meta-analytic approaches. The search yielded 781 articles, with 74 full-text articles reviewed, and 41 studies meeting inclusion criteria. Maternal mental health was a significant individual-level mediator, while child traumatic experiences and insecure maternal–child attachment were consistent family-level mediators. However, the evidence for community-level mediators was limited. A meta-analysis of nine single-mediating analyses from five studies indicated three mediating pathways: maternal depression, negative parenting practices and maternal insecure attachment, with pooled indirect standardised effects of 0.10 [95% CI (0.03–0.17)), 0.01 (95% CI (−0.02 to 0.04)] and 0.07 [95% CI (0.01–0.12)], respectively. Research studies on moderators were few and identified some individual-level factors, such as child sex (e.g. the mediating role of parenting practices being only significant in girls), biological factors (e.g. maternal cortisol level) and genetic factors (e.g. child's serotonin-transporter genotype). In conclusion, maternal depression and maternal insecure attachment are two established mediating pathways that can explain the link between maternal childhood adversity and their children's emotional and behavioural development and offer opportunities for intervention.

## Introduction

Childhood adversity is a serious public health issue with high global prevalence and lifelong negative impact on individuals' health and well-being. Furthermore, its effects cut across generations. Childhood adversity is commonly defined as an exposure to any type of physical abuse, sexual abuse, emotional abuse or neglect that occurred before 18 years of age (Norman et al., 2012). It is estimated that every year millions of children suffer from different forms of abuse and neglect (Afifi & MacMillan, 2011), with a worldwide prevalence ranging between 12.7% and 26.7% (Stoltenborgh, Bakermans-Kranenburg, & Van Ijzendoorn, 2013; Stoltenborgh, Bakermans-Kranenburg, Alink, & Van Ijzendoorn, 2012; Stoltenborgh, Van Ijzendoorn, Euser, & Bakermans-Kranenburg, 2011). As adults, individuals with a history of childhood adversity may be more likely to have children who also experience adversity (Madigan et al., 2019). A history of maternal childhood adversity (MCA, i.e. adversities experienced by mothers when they were children) in particular has been associated with the presence of depression, internalising and externalising problems in their children (Myhre, Dyb, Wentzel-Larsen, Grogaard, & Thoresen, 2014; Su, D'Arcy, & Meng, 2022). This evidence suggests that childhood adversity can lock successive generations of families into poorer health outcomes and a vulnerability to behavioural and mental health problems.

Exploring mediators and moderators in the link between MCA and their children's outcomes is important in clinical prevention, as interventions can precisely target these factors to help improve health outcomes. Mediators are variables that act on the causal pathway between the exposure and outcome, which is influenced by the exposure and in turn influences the outcome, while moderators can affect the direction or strength of the relation between the exposure and the outcome. A significant body of research has investigated the potential mediators underlying the pathway between mothers' experiences of childhood adversity and adverse emotional and behavioural development in their children. These have included maternal individual characteristics, such as maternal mental health problems (Min, Singer, Minnes, Kim, & Short, 2013; Myhre et al., 2014; Plant, Barker, Waters, Pawlby, & Pariante, 2013), and family nurturing factors consisting of maternal hostility (Rijlaarsdam et al., 2014), harsh parenting discipline (Rijlaarsdam et al., 2014; Yoon et al., 2019), maternal social support (Bosquet Enlow, Englund, & Egeland, 2018; Min et al., 2013) and their own children's experience of early adversity (Appleyard, Egeland, van Dulmen, & Alan Sroufe, 2005; Herrenkohl & Herrenkohl, 2007). On the other hand, potential moderators affecting the strength of the relationship between MCA and children's emotional and behavioural development have also been identified. These moderators include maternal mental health (Bouvette-Turcot et al., 2015; Isosavi et al., 2017; Miranda, de la Osa, Granero, & Ezpeleta, 2011), child biological characteristics (Bouvette-Turcot et al., 2015; van de Ven, van den Heuvel, Bhogal, Lewis, & Thomason, 2020; Villani et al., 2018), child sex (Linde-Krieger & Yates, 2018; Yoon et al., 2019) and parenting practices (Meller, Kuperman, McCullough, & Shaffer, 2016; Miranda et al., 2011). However, whether these mediators or moderators consistently play a significant role in the association between MCA and children's emotional and behavioural development remains to be established.

**Fig. 1.**
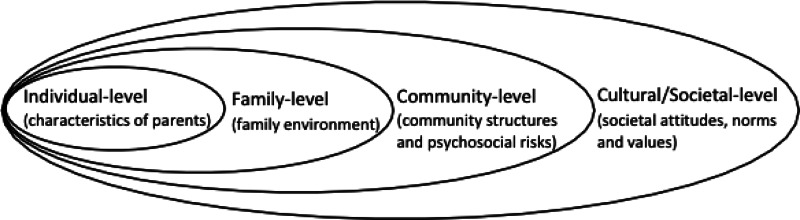
The ecological framework for understanding the link between maternal childhood adversity and their children's mental health development.

To systematically synthesise these factors, we adopted an approach based on the ecological framework of child maltreatment (Belsky, 1980) and on the intergenerational transmission of childhood abuse (Langeland & Dijkstra, 1995) (Fig. 1). This categorises health determinants into four different levels (individual, family, community and societal levels) which can be used to identify vulnerable populations and to inform specific multi-level interventions (Egan, Tannahill, Petticrew, & Thomas, 2008; Sidebotham & Heron, 2006; Wold & Mittelmark, 2018). Since there are no reviews that have comprehensively synthesised or quantified the evidence on the mediators and moderators linking MCA and children's mental health at multiple levels, this review is timely and needed. Evaluating the possible pathways linking maternal adversity and child outcomes with a systematic and quantitative approach would also help delineate the key processes involved, and help identifying the most vulnerable individuals who could benefit from interventions that break the transmission from MCA to adverse outcomes in children.

This review aims to provide a narrative synthesis of existing literature to identify the mediators and moderators that regulate the association between MCA and adverse emotional and behavioural development in children. Furthermore, it aims to investigate the magnitude of any mediating effect by providing a quantitative meta-synthesis.

## Methods

The process of this systematic review and meta-analysis was guided by the Preferred Reporting Items for Systematic reviews and Meta-Analyses (PRISMA) 2020 statement (Yepes-Nuñez, Urrútia, Romero-García, & Alonso-Fernández, 2021). Furthermore, all results were reported based on the categories in an ecological framework (Fig. 1).

### Study search

Systematic searches of peer-reviewed papers in EMBASE, PsycINFO, Medline and the Cochrane Library were conducted up to 26th of February 2021, using a combination of controlled terms and keywords (online Supplementary Appendix A). Systematic searches of grey literature (Adams et al., 2016) were also conducted to identify unpublished articles and documents using search engines, including OpenGrey and WorldCat. Reference lists of included articles were searched and reviewed. Searches were restricted to studies using human participants and written in English, with no restriction on publication year.

**Fig. 2.**
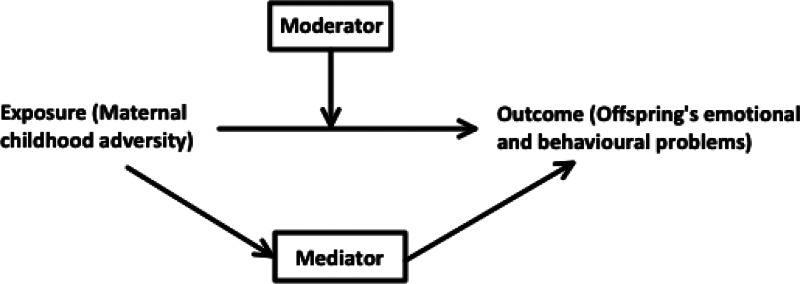
The conceptualisation of the mediator and moderator relationships illustrated by a Directed Acyclic Graph (DAG). Moderator: A variable that affects the direction/or strength of the relation between the exposure and outcome. Mediator: A variable on the causal pathway between the exposure and outcome, which is influenced by the exposure and in turn influences the outcome.

Studies were included if they met the following criteria: (1) used an observational study design (i.e. cohort, cross-sectional and case–control studies); (2) examined the association between a history of childhood adversity in the mother (including biological mothers and step-mothers as the primary caregiver), at least one form of either physical abuse, sexual abuse, emotional/psychological abuse and neglect that occurred before 18 years of age) and their children's emotional and behavioural outcomes [either emotional or behavioural development by age 18 years, including internalising problems, externalising problems, antisocial behaviours, conduct problems, emotional and behavioural dysregulation, depression, anxiety, autism spectrum disorder (ASD) and attention deficit hyperactivity disorder (ADHD)]; and (3) quantitatively analysed at least one mediator or moderator in the association between MCA and children's outcomes (Fig. 2).

We excluded: (1) reviews, meta-analyses, conference abstracts, book chapters or incomplete articles; (2) qualitative studies.

### Screening and data extraction

**Fig. 3.**
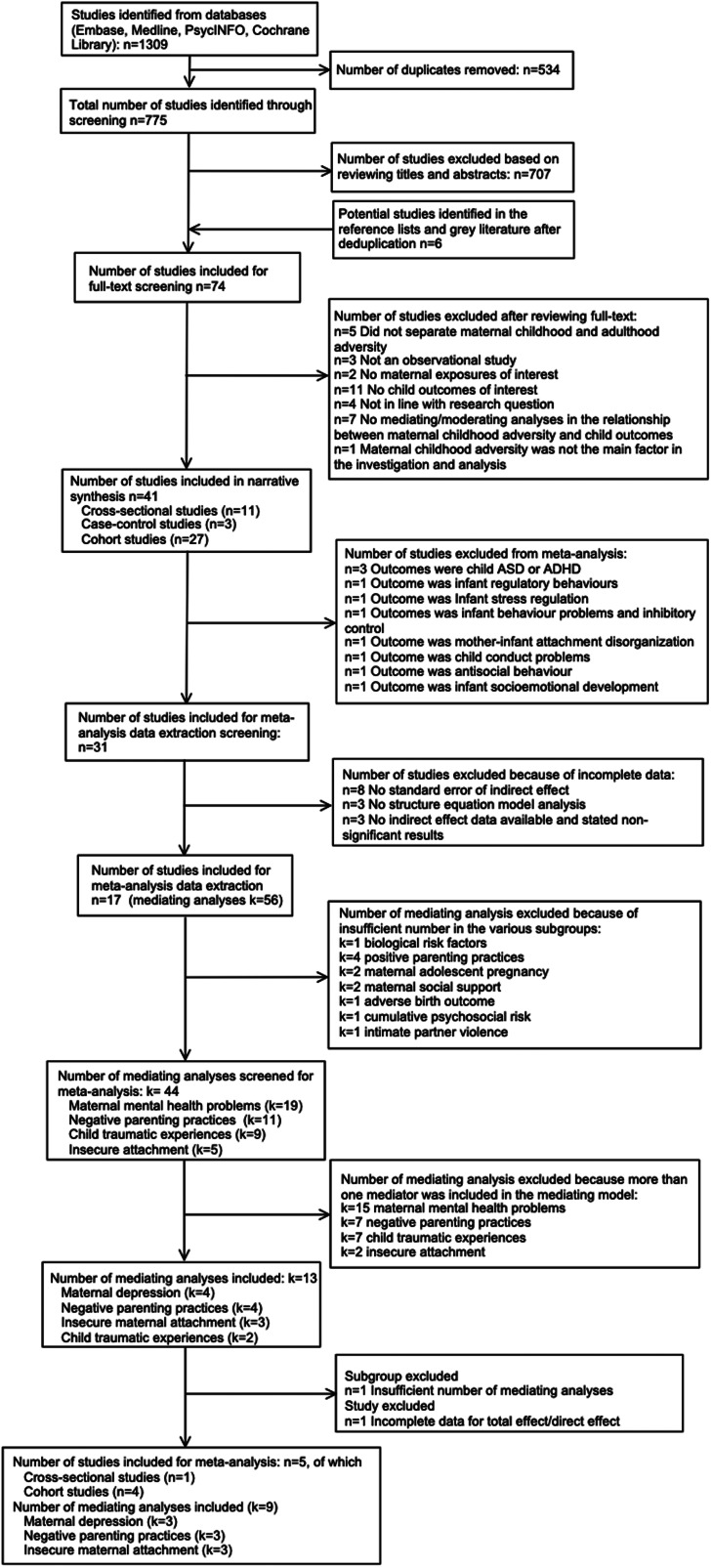
PRISMA flow diagram of the systematic review and meta-analysis processes.

Abstracts and articles for inclusion were double-screened by XM and AB. Upon completion of the initial search, studies were exported to Excel and screened independently by XM and AB to establish reliability and to ensure that no relevant studies were missed. PD and CN were consulted to reach consensus where necessary. For the meta-analysis, effect sizes were extracted by XM, with 10% extracted by CS independently, to ensure accuracy. Effect sizes were indirect standardised effect (*β*) and variance (square of the standard error of *β*) in all mediation models in each study. Where appropriate data were not available, these were calculated using available data in the paper and further requests were sent to study authors. Ultimately, 781 articles' abstracts were screened, and 74 full-text articles were then screened. After review and discussion of discrepancies, 41 studies were selected for the final review and five for the meta-analysis (Fig. 3).

### Strategy for data synthesis

The narrative synthesis provides a detailed account of the samples and study designs, the types and measures of MCA, children's outcomes and measures, the mediating and/or moderating factors in the association between MCA and children's outcomes. We systematically synthesise mediators and moderators at the (1) individual level (maternal characteristics), (2) family level (family relationship, including parenting practices, interaction between parents and child, and domestic violence) and (3) community (psychosocial factors) and societal (societal attitudes and values) level, based on an ecological framework (Fig. 1).

The quantitative meta-analyses were run in R (version 3.6.3), after excluding studies with an insufficient number of consistent outcomes or with incomplete data (Fig. 3). Nine single-mediating analyses were identified and separate meta-analyses were conducted on three mediating pathways: (1) maternal depression; (2) negative parenting practices; and (3) maternal insecure attachment. We used parameter-based meta-analytic structural equation modelling (MASEM) to synthesise the mediation analysis after extracting the indirect effects reported, or computing the indirect effects from the correlation matrices of the primary studies, as well as estimating sampling variances (Cheung, 2022). Afterwards, we used the metaSEM packages to calculate the pooled indirect effects and used restricted maximum likelihood (REML) to estimate parameters in random-effect models (Cheung, 2015). The degree of heterogeneity was assessed by *τ*^2^, which is the heterogeneity variance of the random effects (Borenstein, Higgins, Hedges, & Rothstein, 2017). All *p* values < 0.05 were considered significant.

### Quality assessment

The Newcastle–Ottawa Scales for case–control, cohort and cross-sectional studies (Madhavan, Lagorio, Crary, Dahl, & Carnaby, 2016; Moskalewicz & Oremus, 2020) were used to assess study quality, based on selection, comparability and exposure/outcome (online Supplementary Table S1). A score ⩾7 indicates a good quality study and scores 5–6 indicate a satisfactory quality study (Herzog et al., 2013). Scores ranged from 5 to 8, indicating a satisfactory quality for all studies included in the review (online Supplementary Tables S1–S3).

## Results

Forty-one eligible studies were identified, including 11 cross-sectional studies, 3 case–control studies and 27 longitudinal cohort studies (Fig. 3, Table 1). Most studies were published from 2011 onwards (*n* = 36, 90%), with most conducted in North America (*n* = 25, 61%) and Europe (*n* = 11, 27%), with sample sizes ranging from 45 to 109 758 mother–child dyads. While most studies were population-based, such as the Nurse's Health Study II (Roberts, Liew, Lyall, Ascherio, & Weisskopf, 2018; Roberts, Lyall, & Weisskopf, 2017; Roberts, Lyall, Rich-Edwards, Ascherio, & Weisskopf, 2013), the Avon Longitudinal Study of Parents and Children (ALSPAC) (Roberts, O'Connor, Dunn, Golding, & Team, 2004) and Generation R (Rijlaarsdam et al., 2014), there were also studies from specific populations, including four from children attending psychiatric outpatient services (Bodeker et al., 2019; Miranda et al., 2011, 2013a, 2013b), six from low-income families (Bosquet Enlow et al., 2018; McDonnell & Valentino, 2016; Min et al., 2013; Russotti, Warmingham, Handley, Rogosch, & Cicchetti, 2021; Thompson, 2007; Warmingham, Rogosch, & Cicchetti, 2020), and two including teenage mothers (Pasalich, Cyr, Zheng, McMahon, & Spieker, 2016; Yoon et al., 2019). The most common instruments used to evaluate children's outcomes were the Child Behaviour Checklist (CBCL) (Achenbach, 2011; McConaughy, 2001) (*n* = 19, 46%) and the Strengths and Difficulties Questionnaire (SDQ) (Goodman, 1997) (*n* = 6, 15%), while the most commonly used measure for MCA was the Childhood Trauma Questionnaire (CTQ) (Bernstein, Fink, Handelsman, & Foote, 1998) (*n* = 18, 44%).

**Table 1.** Characteristics of eligible studies included in this review, grouped by study design

Authors, Year	Country	Sample	Maternal exposure (measurement)	Children's outcome (measurement)	Mediators/ Moderators (measurements)	Key findings
*Cross-sectional studies* (*infants*)						
Ludmer et al. (2018)	Canada	*n* = 314; demographically low-risk mother-infant dyads; 67.1% Caucasian, 14.7% Asian 14.7%, 3.1% African–American, 5.9% Hispanic, 9.2% Other ethnic groups.	Maternal childhood maltreatment (CTQ)	Mother-infant attachment disorganisation at age 17 months (Strange Situation Procedure, SSP); rated by trained coders	Individual-level Moderators: Maternal oxytocin receptor (OXTR, rs53576) genotype* (Buccal cells) Maternal cortisol secretion* (Saliva)	Maternal OXTR genotype and maternal cortisol AUCg (area under the curve with respect to ground) moderated the relationship between maternal history of childhood maltreatment and mother-infant attachment disorganisation in the Strange Situation Procedure.
Villani et al. (2018)	Canada	*n* = 193; 72.3% Caucasian, 10.1% Asian, 3.9% African Canadian and 13.6% Other ethnic groups.	Maternal childhood adversity (CTQ)	Infant regulatory behaviours at age 15 months (Toy frustration procedure, TFP); rated by coders	Individual-level Moderator: Infant SLC6A3 and COMT genotypes* (Buccal swabs)	Maternal maltreatment history significantly interacted with infant SLC6A3 and COMT genotypes, such that infants with more than 10-repeat and valine alleles of SLC6A3 and COMT, respectively, relative to infants with fewer or no 10-repeat and valine alleles, utilised more independent regulatory behaviour if the mother reported a more extensive maltreatment history.
*Cross-sectional studies* (*children*)						
Warmingham et al. (2020)	United States	*n* = 378; low-income mothers; 70.5 % Black, 14.6 % White, 10.5 % Hispanic, 4.4 % Other ethnic groups.	Maternal childhood maltreatment (Child Trauma Questionnaire, CTQ).	Child emotion dysregulation at age 10-12 years (Emotion Regulation Checklist); rated by camp counsellors	Individual-level Mediator: **Maternal current depression** (Beck Depression Inventory-II, BDI-II) Family-level Mediator: Child maltreatment* (Maltreatment Classification System in Child Protective Services records)	Maternal childhood maltreatment was associated with both child maltreatment and greater maternal depressive symptoms. Only children's childhood maltreatment mediated the effect of maternal maltreatment on child emotion dysregulation, rather than maternal depression.
Bodeker et al. (2019)	Germany	*n* = 194; part of a large multicentre study in clinical settings (Understanding and Breaking the Intergenerational Cycle of Abuse, UBICA); Majority were German (90.7%).	Maternal childhood maltreatment (CECA interview)	Child psychopathology at age 5-12 years (CBCL); rated by parents and teachers	Family-level Mediator: **Maternal sensitivity** (Emotional Availability Scales)	Path analyses showed that maternal sensitivity mediated the effect of a maternal history of depression on parents' ratings of child psychopathology. In contrast, maternal childhood maltreatment was directly linked to teachers' ratings of child psychopathology and this effect was not mediated by maternal sensitivity.
Meller et al. (2016)	United States	*n* = 64; 50.0% African–American, 45.3 White non-Hispanic, 1.6% Hispanic, and 3.1% Multi-ethnic groups.	Maternal childhood adversity (CTQ)	Child behaviour problems at age 8-11 years (CBCL); rated by parents	Individual-level Mediator: Maternal current depressive symptoms* (Symptom: Checklist-90-Revised, SCL-90-R) Family-level Moderator: Maternal hostility* (Parent–child interaction task)	Maternal depressive symptoms mediated the relationship between maternal history of emotional maltreatment and child's behaviour problems. In addition, this mediating effect was strongest in the presence of high levels of maternal hostility (i.e. + 1 s.d.).
Miranda et al. (2013a)	Spain	*n* = 327; children from psychiatry outpatient services; 97.9% Caucasian.	Maternal childhood psychological, physical, and sexual abuse (Structured interview with Schedule of Risk Factors, SRF)	Child externalising behaviour at age 8-17 years (CBCL); rated by mothers Children's functioning (Child and Adolescent Functioning Assessment Scale, CAFAS); rated by interviewer	Individual-level Mediators: Maternal current depressive symptoms* (SCL-90-R) **Maternal anxiety** (SCL-90-R) Family-level Mediator: **Maternal hostility** (SCL-90-R) Individual-level Moderator: **Child sex**	Maternal depressive symptoms were the main mediator in the relationship between maternal childhood adversity, intimate partner violence and children's externalising problems. Children's sex did not have a moderating role in adjusted paths.
Miranda et al. (2013b)	Spain	*n* = 318; Psychiatric outpatient services; 98.1% Caucasian.	Maternal childhood adversity (SRF)	Child externalising and total behaviour problems at age 8-17 years (CBCL); rated by mothers Children's functioning (CAFAS); rated by interviewer	Individual-level Mediators: Maternal current depression* (SCL-90-R) Family-level Mediators: Child negative life events* (Life Events Checklist) **Physical punishment** (Parental Discipline Practices Scales) **Maternal rejection** (EMBU, Parental style)	Mothers' depression mediated the link between maternal childhood adversity, intimate partner violence, cumulative violence and children's externalising, and total behaviour problems. Children's negative life events were important factors in the link between maternal childhood adversity and total behaviour problems, and between cumulative violence and both externalising and total behavioural problems.
Esteves et al. (2017)	United States	*n* = 101; predominantly in high-risk neighbourhoods and of African–American ethnicity.	Maternal physical maltreatment (Adverse Childhood Experiences Study Questionnaire)	Child internalising behaviour at age 5-16 years (CBCL); rated by mothers	Individual-level Mediator: **Maternal current depressive symptoms** (CES-D) Family-level Mediators: **Parenting practices** (CTS-PC) **Child's exposure to stressful life events** (Post Traumatic Stress Disorder Module of the Preschool Age Psychiatric Assessment, PAPA)	Maternal childhood exposure to physical maltreatment was significantly associated with child's internalising symptoms; this effect remained after accounting for child sex, maternal depressive symptoms, harsh parenting practices, and the child's own exposure to stressful life events. Formal tests of mediation through these pathways were non-significant.
Miranda et al. (2011)	Spain	*n* = 547; Psychiatry outpatient settings; predominantly Caucasian.	Maternal childhood abuse (SRF)	Children's internalising and externalising problems at age 8-17 years (CBCL); rated by parents	Individual-level Mediators and Moderators: **Maternal historical mental problems** (Family Psychiatric History Screen for Epidemiologic Studies) **Father's historical mental health** Family-level Mediator and Moderator: **Physical punishment of children** (Parental Discipline Practices Scales)	Parents' psychopathology and physical punishment of children did not act as moderators or mediators.
Oshio and Umeda (2016)	Japan	*n* = 1003 parents and *n* = 1750 children; Japanese Study of Stratification, Health, Income, and Neighbourhood (J-SHINE).	Mother's childhood physical abuse and neglect [Reported answers (yes or no) to questions about experiences before age 15]	Children's problem behaviour at age 2-18 years (CBCL); rated by parents	Individual-level Mediator: Parents' current psychological anxiety* (Kessler Psychological Distress Scale, K6)	The impact of maternal childhood abuse on daughters' problem behaviour was mediated by both parents' psychological distress. Strong mother-daughter and father-son linkages were observed: daughters' problem behaviour was more closely associated with mothers' than fathers' childhood abuse, whereas sons' problem behaviour was more closely associated with their fathers' experience.
Russotti et al. (2021)	United States	*n* = 378, economically disadvantaged; 70.5 % Black, 10.5 % Hispanic, 14.6 % white, 4.4 % Other ethnic groups.	Maternal childhood maltreatment (CTQ)	Child's internalising and externalising symptoms at age 10-12 years (Pittsburgh Youth Survey, PYS; CBCL-TRF; rated by counsellors; Children's Depression Inventory, CDI; self-report; Revised Children's Manifest Anxiety Scale, RCMAS; self-report)	Individual-level Mediators: Maternal current depressive symptoms* (BDI-II); **Maternal adolescent childbearing** (Mothers who began childbearing when they were < 20-years-old) Family-level Mediators: Chronic childhood maltreatment* (Child Protective Services (CPS) records);	Maternal maltreatment indirectly affected child internalising symptoms through chronic childhood maltreatment and maternal depressive symptoms, but not via maternal adolescent childbearing. Maternal history of child maltreatment had an indirect effect on Child externalising symptoms through children's childhood maltreatment, but not through adolescent childbearing or maternal depression.
*Case*–*control studies* (*children*)						
Roberts et al. (2013)	United States	*n* = 52 949 (451 mothers of children with autism and 52 498 mothers of children without autism); Nurses' Health Study II; 97.0% White ethnic group.	Maternal childhood physical and emotional abuse < age 12 years combined (CTQ) Maternal childhood sexual abuse < age 12 years and age 12–17 years	Child ASD (the Autism Diagnostic Interview-Revised); rated by diagnosis	Individual-level Mediators: Gestational diabetes* Abortion history* Smoking* Family-level Mediator: **Intimate partner** (Modified version of the Assessing Abuse Scale) Individual-level Moderator: **Child sex**	Gestational diabetes (mediation, 3.5%) and abortion before parturition (mediation, 3.0%) were the strongest mediators of the relationship between child abuse and autism in children. Smoking during pregnancy mediated 2.3% of the association. An abuse × sex interaction term was not statistically significant.
Roberts et al. (2017)	United States	*n* = 209 mothers of children with autism and n = 833 mothers of children without autism; Nurses' Health Study II; 97.7%. White, non-Hispanic.	Maternal childhood sexual abuse < age 12 and age 12–17 years, and physical and emotional abuse < age 12 (CTQ)	Child autism spectrum disorder (ASD) case status (Autism Diagnostic Interview-Revised); rated by diagnosis	Individual-level Mediator: Maternal and paternal autistic traits* (Social Responsiveness Scale, SRS)	Maternal and paternal autistic traits accounted for 21% of the association between maternal abuse and their children's autism.
Roberts et al. (2018)	United States	*n* = 49 497 mothers and n = 7607 children with ADHD, n = 102 151 control children; Nurses' Health Study II cohort; Predominantly White ethnic group.	Maternal childhood physical and emotional abuse (CTQ) Sexual abuse up to age 11 years and ages 11–17 years (Parent-Child Conflict Tactics Scales)	Attention deficit hyperactivity disorder (ADHD) (ADHD Rating Scale-IV); rated by diagnosis and maternal report	Individual- and family- level Mediators: **Adverse perinatal circumstances** (e.g. Prematurity, Smoking, Gestational diabetes, Intimate partner violence) Community-level Mediators: **Socioeconomic factors** (e.g. Education, Family income, Marital status)	The association between maternal experience of childhood abuse and risk for ADHD in their children was not explained by perinatal risk factors or socioeconomic status.
*Cohort studies* (*infants*)						
Bouvette-Turcot et al. (2015)	Canada	*n* = 154; 88.7% European/Caucasian, 8.1% African descent/African–American, and 3.2% Hispanic/Latino.	Maternal childhood adversity (CTQ) Parental Bonding Instrument (PBI) Principal component analysis was used to derive one factor (CTQ and PBI)	Infant negative emotionality/behavioural dysregulation (NE/BR) at age 18-36 months (Early Childhood Behaviour Questionnaire); rated by mothers Emotional- behavioural functioning at age 60 months (SDQ); rated by parents	Individual-level Moderators: Child 5-HTTLPR genotype* (A functional promoter polymorphism in the serotonin-transporter gene (SLC6A4, Buccal swabs) **Maternal postpartum depression** (Centre for Epidemiologic Studies Depression Scale, CES-D).	There was a significant interaction effect of maternal childhood adversity and their children's 5-HTTLPR genotype on child negative emotionality/behavioural dysregulation. Children with the less functional 5-HTTLPR (S/LG allele carriers) alleles had significantly higher NE/BR scores than LA/LA homozygotes at high levels of maternal adversity but had significantly lower NE/BR scores at low levels of maternal adversity.
Liu et al. (2019)	China	*n* = 207; community sample; 100% Chinese.	Maternal childhood emotional abuse, CEA (CTQ-SF)	Infant behaviour problems and inhibitory control (IC) at age 14 months (Infant-Toddler Social and Emotional Assessment, ITSEA and a reverse categorisation task); rated by experimenters	Family-level Mediator: Maternal negative expressiveness* (Self-Expressiveness in the Family Questionnaire, SEFQ). Individual-level Moderator: Infant inhibitory control* (The age-appropriated simplified version of the reverse categorisation task).	Maternal negative expressiveness significantly mediated the positive relation between maternal CEA and infant externalising, internalising and dysregulation problems. In addition, the mediating pathway from maternal CEA to dysregulation problems through maternal negative expressiveness was significant, but only in infants with poor IC. The results were robust even after controlling for family socio-economic status, and maternal childhood physical and sexual abuse.
Isosavi et al. (2017)	Palestine	*n* = 511; from 10 maternal clinics in government primary healthcare centres (PHCC) in the Gaza Strip; Palestinian women.	Maternal childhood physical and emotional abuse before age 12 years (13-item questionnaire developed by the Transcultural Psychosocial Organization)	**Infant** stress regulation at age 4 months (The Infant Behaviour Questionnaire- Revised (IBQ-R, short version); rated by parents Infant's typical behaviour at age 4 months (The 91-item questionnaire); rated by parents	Individual-level Mediator: **Maternal antenatal mental symptoms** (Edinburgh Depression Scale (EDS); Perceived Stress Scale; Harvard Trauma Questionnaire) Individual-level Moderator: Maternal war trauma* (Traumatic events common and typical during the 2008 to 2009 Gaza War and the 2012 military offensive)	Maternal CEA predicted negative affectivity (in terms of infant stress regulation), but only among mothers with low war trauma. However, the effects of maternal trauma on infant stress regulation were not mediated by mental health symptoms. Maternal higher socio-economic status was associated with better infant stress regulation, whereas infant prematurity and male sex predisposed to difficulties.
Choi et al. (2017)	South Africa	*n* = 150; community sample; predominantly mixed race.	Maternal childhood adversity (CTQ)	Symptoms of emotional and/or behavioural difficulties at age 1 year (**Infant**/Toddler Symptom Checklist, ITSCL); rated by mothers	Individual-level Mediator: Postpartum depression* (EPDS) Family-level Mediator: Maternal affective processing* (Modified Stroop task)	Postpartum depression was a significant mediator, and the effect persisted for maternal-infant bonding and infant growth after controlling for covariates and antenatal distress.
McDonnell and Valentino (2016)	United States	*n* = 398; from Women, Infants, and Children health clinics. 30.4% African–American, 53.3% Caucasian, and 16.3% Other ethnic groups.	Maternal childhood maltreatment and household dysfunction (Family health history questionnaire (FHHQ), female version)	**Infant** socioemotional functioning at age 6 months (Ages and stages questionnaire-socioemotional, ASQ-SE); rated by mothers	Individual-level Mediator: **Maternal depressive symptoms differences between prenatal and 6 months postpartum**	Maternal depressive symptoms did not mediate the association between maternal childhood maltreatment and infant socioemotional functioning.
*Cohort studies* (*children*)						
Giallo et al. (2020)	Australia	*n* = 1507; first-time mothers from Maternal Health Study; 71.3% born in Australia.	Maternal childhood physical or sexual abuse (Maltreatment History Self Report)	Children's emotional-behavioural functioning at age 10 years (SDQ); rated by parents	Individual-level Mediators: Maternal postpartum depressive symptoms at 12 months* (Edinburgh Postnatal Depression Scale, EPDS) **Adverse birth outcomes** (Pre-term birth and/or Low birthweight) Family-level Mediator: Postpartum exposure to intimate partner violence* (Short 18 item Version of the Composite Abuse Scale, CAS).	Psychosocial health pathways via maternal depressive symptoms and exposure of mothers to intimate partner violence in the first 12 months postpartum, but not adverse birth outcomes, mediated the association between maternal childhood abuse and children's emotional and behavioural difficulties.
Collishaw et al. (2007)	United Kingdom	*n* = 5619; the Avon Longitudinal Study of Parents and Children (ALSPAC) cohort; Predominantly White ethnic group.	Maternal childhood physical and emotional abuse prior to age 17 and sexual abuse prior to age 16. Self-report.	Children's adjustment at age 4-7 years (SDQ); rated by parents Children's adjustment at age 7 years (SDQ); rated by teachers	Individual- and family- level Mediators: Antecedent psychosocial factors* (Maternal antenatal depression, Anxiety, and Somatic complaints at 32 weeks (Crown-Crisp Experiential Index, CCEI); Maternal hostility to the child (Parent–child relationship); Family type; Earlier family stressful events) Family-level Mediator: Interim stressful events* (Child Life Events)	Interim life events, together with antecedent psychosocial risk (maternal antenatal affective symptoms, age 4 parental hostility, age 4 family type) fully mediated the association between maternal childhood abuse and their children's prognosis.
Bosquet Enlow et al. (2018)	United States	*n* = 187; low-income families; 80% White, 13% Black, 5% Native American, 1% Asian, and 1% Hispanic.	Quality of care received by mothers, including living conditions; feelings toward their parents; degree of emotional support; discipline methods; and exposure to neglect, physical abuse, and/or sexual abuse (Interview)	Child emotional and behavioural problems at age 7 years (CBCL); rated by mothers and teachers	Individual-level Mediator: **Maternal stress at 18–42 months postpartum** (Life Event Scale) Family-level Mediators: Child maltreatment* (Home and videotaped laboratory observations) **Maternal caregiving quality** (Videotaped laboratory tasks; Supportive Presence Scale; The Hostility Scale) Community-level Mediator: **Social support** (Support scale)	A path analysis model showed a mediation effect of maternal childhood maltreatment on child symptoms, with a specific effect for child maltreatment. A history of maternal maltreatment was associated with stress exposures and social support during both developmental periods, even after accounting for the association between maternal history and child maltreatment.
Linde-Krieger and Yates (2018)	United States	*n* = 225; 56.9% Latina, 17.3% Black, 20% White, and 5.8% Multiracial/other.	Maternal sexual abuse prior to age 18 (Structured interview)	Children's externalising and internalising behaviour problems at age 4 years and 8 years (Test Observation Form, TOF); rated by examiners	Family-level Mediator: Helpless state of mind*(Caregiving Helplessness Questionnaire, CHQ at Age 6). Individual-level Moderator: Child sex*	It revealed a small-to-medium indirect effect of mothers' childhood sexual abuse severity on child externalising problems, through increased maternal helplessness for girls, but not for boys.
Pereira et al. (2018)	Canada	*n* = 96; community sample from a longitudinal study; 82.3% Caucasian.	Maternal childhood maltreatment (The Childhood Trauma Questionnaire Short Form, CTQ-SF)	Child behaviours at age 5 years (CBCL); rated by mothers	Individual-level Mediator: Maternal depressive symptoms at 16 months postpartum* (BDI-II**);** Family-level Mediator: Avoidant attachment* (Experience in Close Relationships Inventory (ECR)); Community-level Mediator: **Social support** (Multidimensional Scale of Perceived Social Support, MSPSS).	Only maternal depressive symptoms mediated the relation between maternal maltreatment history and children's internalising problems. With respect to the relationship between maternal maltreatment history and children's externalising problems, only maternal depressive symptoms and avoidant attachment accounted for unique mediating variance.
Choi et al. (2019)	United Kingdom	*n* = 1016 mothers and their n = 2032 children; Environmental Risk (E-Risk) Longitudinal Twin Study; Around 90% White.	Maternal childhood adversity (CTQ)	Child internalising and externalising symptoms at age 12 years (CBCL); rated by mothers and teachers	Individual-level Mediators: Maternal later cumulative depression* (Interviewed by a trained clinician using the standardised Diagnostic Interview Schedule based on DSM-IV criteria) **Maternal postpartum depression up to 5 years** (Diagnostic Interview Schedule and Life History Calendar) Family-level Mediator: Child maltreatment* (Exposure to physical and sexual maltreatment by an adult) Individual-level Moderator： **Child sex**	Indirect effects of maternal childhood maltreatment on children's outcomes were robust across child sexes and supported a significant mediating effect for postpartum depression; however, this appeared to be carried by maternal depression beyond the postpartum period.
Madigan et al. (2017)	Canada	*n* = 501; community mother-infant dyads; 56.5% White.	Maternal reporting of family dysfunction and victimisation before the age of 16 years, and sexual and physical victimisation (Childhood Experience of Violence Questionnaire)	Children's emotional problems at age 18 months (Scales adapted for use in the National Longitudinal Survey of Children and Youth); rated by parents	Individual-level Mediator: Maternal postpartum depression at 2 months (clinical level CES-D ⩾ 16) Community-level Mediators: Cumulative psychosocial risk* (Single parent; Teenage mother; Low family income (<$ 20 000); Low maternal education (⩽ high school); Marital conflict)	The relationship between adverse childhood experiences and infant emotional health operated specifically through cumulative psychosocial risk.
Yoon et al. (2019)	United States	*n* = 495; teen mothers; 58.2% White, 25.8% African–American, 6.7% Native American, 4.4%, Asian, and 5.0% Other ethnic groups.	Maternal childhood adversity before age 18 (8 items, including physical and sexual abuse)	Externalising behaviour at age 11 years (CBCL); rated by parents	Family-level Mediators: Physical discipline* (Conflict Tactics Scale) Parenting stress* (Parenting Stress Index, PSI) Individual-level Moderator: Child sex*	The path between physical discipline and externalising behaviour differed by gender, with the path being only significant for girls.
Plant et al. (2017)	United Kingdom	*n* = 9397; 96.7% White.	Maternal childhood maltreatment (physical, sexual, and emotional abuse and neglect, <18 years)	Child's preadolescent emotional and behavioural difficulties (Development and Well-Being Assessment (DAWBA); at age 10 years and 8 months, and at age 13 years 10 months; SDQ at age 11 years 8 months); rated by mothers	Individual-level Mediators: Maternal antenatal depression at 18-32 weeks of pregnancy* (EPDS) Postnatal depression at 8 weeks and 8 months postpartum* (EPDS) Family-level Mediators: Child childhood maltreatment* (Bullying and Friendship Interview Schedule) **Maladaptive parenting** (Self-report)	Maternal antenatal depression, postnatal depression and their children's childhood maltreatment, but not maladaptive parenting, significantly and independently mediated the association between maternal child maltreatment and both internalising and externalising difficulties.
Pasalich et al. (2016)	United States	*n* = 112; teen mother; 78.6% White, 9.8% African–American, 5.4% Native American, 1.8% Hispanic/Latina, and 4.5% Mixed heritage.	Maternal physical and sexual abuse history (home interview)	Child externalising problems at age 4.5 and 9 years (CBCL); rated by mothers	Family-level Mediators: Infant insecure attachment* (Strange Situation Procedure) **Maternal hostility** (Parent–Child Interaction Task)	Compared to teen mothers reporting no abuse history, teen mothers with a history of sexual and physical abuse were more likely to have an infant with an insecure attachment, which predicted elevated externalising problems in preschool age, which in turn was associated with subsequent externalising problems.
Madigan et al. (2015)	Canada	*n* = 490; 56.5%, Caucasian, 14.6% South Asian, 12.0% Black, and 7.7% Other ethnic groups.	Maternal childhood physical and sexual abuse <16 years (Adapted version of the Childhood Experience of Violence Questionnaire, CEVQ)	Children's internalising problems at age 36 months (adapted scales for use in the National Longitudinal Survey of Children and Youth); rated by parents	Individual-level Mediator: Maternal postpartum depression at 2 months after childbirth* (CES-D) Family-level Mediator: **Responsive Parenting** (Three 5-min tasks: an unstructured, free-play task; a structured, cooperative teaching task; and a wordless picture book task)	There was a significant indirect effect of maternal physical abuse on children's internalising problems through maternal depressive symptoms.
Myhre et al. (2014)	Norway	*n* = 25 452; Population-based pregnancy cohort; 95% Norwegian and Scandinavian ethnicities.	Maternal childhood adversity (Four items: (1) degradation or humiliation, (2) threats, (3) physical abuse, (4) sexual abuse as a child (<18) or an adult, based on the Norvold Abuse Questionnaire, NorAq)	Child externalising behaviour at age 36 months (CBCL); rated by mothers	Individual-level Mediator: Maternal postpartum mental distress at 18 months* (Anxiety and Depression, the Symptom Checklist, SCL-8)	Increased maternal mental distress partly accounted for the relationship between maternal childhood abuse and increased externalising behaviour in the children and was a partial mediator in the relationship.
Min et al. (2013)	United States	*n* = 231; mothers at high risk because of drug use, and from primarily poor, urban settings; 81% African–American ethnicity.	Maternal childhood trauma (CTQ)	Child behaviour problems at age 9 years (CBCL); rated by parents and children	Individual-level Mediator: Maternal postpartum psychological distress at 6 years* (Somatic complaints, Obsessive-compulsive behaviour, Interpersonal sensitivity, Depression, Anxiety, Hostility, Phobic anxiety, Paranoid ideation, and Psychoticism; the Brief Symptom Inventory, BSI) Community-level Mediator: Social support* (MSPSS); Individual-level Moderator: Child sex	Maternal social support was a mediator of child self-reported behaviour, and maternal psychological distress was a mediator of maternal report of child behaviour. No significant gender interaction was found in the gender-specific model.
Plant et al. (2013)	United Kingdom	*n* = 125; from the South London Child Development Study; 72% White ethnicity.	Maternal childhood physical abuse, sexual abuse, emotional neglect and physical neglect (maltreatment was rated if two or more types of maltreatment were reported)	Adolescent antisocial behaviours (DSM-IV symptoms of disruptive behaviour disorders (DBDs) were recorded from combined (parent and child) psychiatric interview reports at age 11 and 16 years using the Child and Adolescent Psychiatric Assessment (CAPA))	Family-level Mediator: Child maltreatment* (The combined (parent and child) 11-year Child and Adolescent Psychiatric Assessment, CAPA) Individual-level Moderator: Maternal antenatal depression* (Clinical Interview Schedule, CIS)	Children's exposure to childhood maltreatment played a mediating role in the link between maternal psychosocial adversity and their children's antisocial behaviour, and the pathway was significant only in the children of mothers with a history of antenatal depression.
Thompson (2007)	United States	*n* = 197 mother–child dyads from LONGSCAN; low-income neighbourhoods; 58.4% African–American, 20.8% White, 14.7% Hispanic, and 6.1% mixed race.	Maternal early life victimisation (maternal history of victimisation, mainly physical, developed by LONGSCAN staff to assess caregivers' history of loss and victimisation)	Child behaviour problems at age 4 years (CBCL); rated by caregivers	Individual-level Mediator: **Maternal postnatal psychosocial functioning** (CES-D; CAGE Alcohol Abuse Screening) Family-level Mediator: Maternal psychological aggression* (Conflict Tactics Scale-Parent-Child, CTS-PC) Community-level Mediators: **Socio-economic factors** (Family income, Maternal education, Age at child's birth, and Marital status)	Mothers' early experiences with violence victimisation influenced child behavioural outcomes, which was partly mediated by mothers' psychological aggression toward their children.
Zvara et al. (2017)	United States	*n* = 204; living in poor, rural communities; Longitudinal design from Family Life Project (FLP); 56,4 % European Americans and 43.6 % African–Americans.	Maternal sexual trauma at or before the age of 14 years (Trauma History Questionnaire, THQ)	Child Conduct Problems at Grade 1 (SDQ); rated by mothers	Individual-level Mediator: Maternal depressive symptoms at 6-, 15-, 24-, and 36-months postpartum* (BSI) Family-level Mediators: Intimate partner violence* (CTS-R) Maternal sensitive parenting* (Two tasks were observed and coded by researchers)	After controlling for numerous sociodemographic factors, the analyses indicated that maternal depressive symptoms, intimate partner violence, and maternal parenting were significant mediators.
Rijlaarsdam et al. (2014)	Netherland	*n* = 4438, Generation R; 24.9% non-Western origin (10.0% Mediterranea*n*, 8.3% Caribbean, and 6.6% other non-Western).	Maternal childhood maltreatment (CTQ)	Children's internalising and externalising problems at age 6 years (CBCL); rated by parents and children	Family-level Mediators: Maternal hostility* (BSI) Maternal harsh discipline* (CTS-PC) Fathers' hostility* (Same as above) Fathers' harsh discipline* (Same as above)	Maternal maltreatment was indirectly associated with both parental and child reports of externalising problems through maternal hostility and maternal harsh discipline, and through fathers' hostility and fathers' harsh discipline.
Roberts et al. (2004)	United Kingdom	*n* = 8292; ALSPAC; 9.0% Single mother families, 79.5% Biological families, 4.6% Stepmother/complex stepfamilies, and 6.9%, Stepfather families.	Maternal childhood sexual abuse, CSA (Self-report data on prior sexual assault)	Child adjustment at age 47 months (SDQ); self-report	Individual-level Mediators： **Maternal postpartum depression at 33 months** (EPDS) Maternal postpartum anxiety at 33 months* (Crown-Crisp Experiential Index) Family-level Mediator: Maternal bonding* (Mother's enjoyment of, and Confidence in her relationship with her child)	Maternal CSA was associated with later adjustment in their children, partially mediated by maternal mental health (principally) and maternal confidence in maternal–child relationship. The link between CSA and later maternal confidence was also partially mediated by maternal mental health.
Bouvette-Turcot et al. (2020)	Canada	*n* = 239; Maternal Adversity, Vulnerability, and Neurodevelopment (MAVAN); 88.7% European/Caucasian, 8.1% African descent/African–American, and 3.2% Hispanic/Latino ethnicities.	Maternal childhood adversity (CTQ and PBI）	Child negative emotionality/behavioural dysregulation at age 36 months (Early Childhood Behaviour Questionnaire, ECBQ); rated by mothers	Individual-level Mediator: Maternal postpartum depression at 6 months * (EPDS) Family-level Mediator: Maternal sensitivity* (Ainsworth maternal sensitivity scale)	Maternal depression mediated the effect of maternal childhood adversity on child negative emotionality/behavioural dysregulation. Also, there was a serially indirect effect of maternal childhood adversity on child negative emotionality/behavioural, mediated first by maternal depression and then by maternal sensitivity.
Roth et al. (2021)	United States	*n* = 96; 60.4% White and 19.8% Asian.	Maternal childhood maltreatment (CTQ)	Child's emotional and behavioural problems at age 18–72 months (CBCL); rated by mothers	Family-level Mediator: Maternal attachment style* (The Attachment Style Questionnaire, ASQ)	Less secure maternal attachment, but not avoidant or anxious attachment, mediated the association between maternal childhood maltreatment and their children's emotional and behavioural problems.
van de Ven et al. (2020)	United States	*n* = 45; 77.8% African–American/Black， 13.3% Caucasian/White， 2.2% Asian, and 6.7% Other ethnic groups.	Maternal childhood trauma (CTQ)	Child's emotional and behavioural problems at age 5 years (CBCL); rated by mothers	Individual-level Mediator: **Frontal alpha asymmetry, FAA** (Electroencephalography, EEG) Individual-level Moderator: FAA* (EEG)	FAA was not significantly associated with maternal childhood trauma or child total and externalising behavioural problems. In contrast, FAA did moderate the relationship between maternal childhood trauma and total and externalising behavioural problems.
Linde-Krieger and Yates (2021)	United States	*n* = 250; pre-schoolers and their female caregivers from an ongoing longitudinal study; 46% Hispanic, 18% Black, 11.2% white, 0.4% Asian, and 24.4% multiracial.	Maternal childhood trauma (Early Trauma Inventory)	Child emotional and behavioural problems (Diagnostic Interview Schedule for Children – IV, C-DISC at age 10 years) and TOF at age 4 years); self-report and rated by examiners	Family-level Mediators: **Maternal negative parenting** (Child's negative representation of the parent) **Parent-child role confusion**	There was a significant indirect effect of parents' severity of child maltreatment on child psychopathology through parent-child role confusion and children's negative representations of the parent, indicating serial mediation, but non-significant single pathways.

*Note*: *Significant factors.

### Mediators

#### Individual-level mediators

Individual-level mediators included maternal depression, anxiety, and perinatal health.

The mediating role of maternal mental health was reported in several studies, with more evidence for a role of depression than anxiety. We identified 10 longitudinal studies that reported that children of mothers exposed to childhood adversity were more likely to develop emotional-behavioural problems than children of mothers who were not exposed to MCA, with 6–45% of the total effects being mediated by postnatal depression (Bouvette-Turcot et al., 2020; Choi et al., 2017; Giallo et al., 2020; Madigan, Wade, Plamondon, & Jenkins, 2015; Madigan, Wade, Plamondon, Maguire, & Jenkins, 2017; Min et al., 2013; Myhre et al., 2014; Pereira, Ludmer, Gonzalez, & Atkinson, 2018; Plant, Jones, Pariante, & Pawlby, 2017; Zvara, Mills-Koonce, Carmody, & Cox, 2017). Of note, one study reported that both maternal antenatal and postnatal depression independently mediated the relationship between MCA and children's emotional-behavioural problems, with 20 and 6% of the total effects on child internalising behaviours, and 14 and 37% on child externalising behaviours being explained by antenatal and postnatal depression respectively (Plant et al., 2017). On the other hand, four cross-sectional studies suggested that current maternal depression positively fully or partially mediated the pathway from MCA to child problems, such as anxiety and depression, and rule-breaking and aggressive behaviours (Meller et al., 2016; Miranda, de la Osa, Granero, & Ezpeleta, 2013a, 2013b; Russotti et al., 2021), and one study suggested that maternal depression throughout life, rather than postnatal depression, played a mediating role beyond the postpartum period (Choi et al., 2019).

Only two studies assessed maternal anxiety as an independent mediator and three included maternal anxiety in the assessment. Among these, three indicated that maternal mental distress, including anxiety significantly mediated the association between MCA and the presence of children's internalising and externalising problems (Min et al., 2013; Myhre et al., 2014; Oshio & Umeda, 2016), and the UK ALSPAC study suggested that maternal postnatal anxiety rather than depression independently mediated 16% of the relationship between maternal childhood sexual abuse and poorer child emotional-behavioural adjustment at age 47 months (Roberts et al., 2004). However, another cross-sectional study conducted among psychiatric outpatient children in Spain found the opposite result, suggesting that maternal postpartum depression rather than anxiety mediated the relationship between MCA and child externalising behaviour in 8- to 17-year-old youths (Miranda et al., 2013a). It is possible that maternal anxiety is associated with mental health problems in young children, while maternal depression could have more influence on adolescent mental health.

By contrast, seven studies did not support the presence of a mediating role for any type of maternal mental health problems in the relationship between MCA and child (Bosquet Enlow et al., 2018; Esteves, Gray, Theall, & Drury, 2017; Miranda et al., 2011; Thompson, 2007; Warmingham et al., 2020) or infant emotional-behavioural problems (Isosavi et al., 2017; McDonnell & Valentino, 2016). Interestingly, six of these studies included participants from low-income or high-risk neighbourhoods, suggesting that lower socioeconomic circumstances may modify the mediating effect of maternal mental health problems.

Only four studies investigated the role of maternal perinatal health as a mediator. One study suggested that gestational diabetes, abortion history and smoking during pregnancy significantly mediated 3.5, 3.0 and 2.5% respectively, of the association between MCA and an increased risk of their children's autism (Roberts et al., 2013), while another study did not find perinatal risk factors to be mediators for ADHD (Roberts et al., 2018). Furthermore, maternal adolescent childbearing, their children's preterm birth or low birth weight were not significant mediators in the relationship between MCA and children's emotional-behavioural difficulties (Giallo et al., 2020; Russotti et al., 2021).

#### Family-level mediators

Family-level mediators included child traumatic experiences, negative and positive parenting practices, maternal–child attachment, and intimate partner violence.

Notably, traumatic experiences in the children themselves have been posited as mediators in the link between MCA and child emotional-behavioural problems, with eight out of nine studies that investigated this factor reporting its significant role in mediating 17–47% of the total effects (Bosquet Enlow et al., 2018; Choi et al., 2019; Collishaw, Dunn, O'Connor, & Golding, 2007; Miranda, de la Osa, Granero, & Ezpeleta, 2013b; Plant et al., 2013; Plant et al., 2017; Russotti et al., 2021; Warmingham et al., 2020). Only one cross-sectional study, conducted among 101 predominantly African–American mother–child dyads living in high-risk neighbourhoods in the United States, did not report this link (Esteves et al., 2017), suggesting that socioeconomic deprivation may have attenuated the mediating effect of child traumatic experiences.

Parenting practices, both negative and positive, have been the most frequently investigated family-level mediators in the relationship between MCA and children's emotional-behavioural problems. Evidence is inconsistent for the role of negative parenting practices, with two longitudinal population-based studies indicating these significantly mediated 15–70% of the total effect (Rijlaarsdam et al., 2014; Yoon et al., 2019). However, two studies in psychiatric outpatient settings, and one in a deprived community sample did not find any effect for harsh parenting discipline (Esteves et al., 2017; Miranda et al., 2011, 2013b). Five studies investigated the potential mediating effect of maternal hostility as a negative parenting mediator. Of these, two suggested this played a significant mediating role (Collishaw et al., 2007; Rijlaarsdam et al., 2014), while the other three studies did not (Bosquet Enlow et al., 2018; Miranda et al., 2013a; Pasalich et al., 2016). Of note, these studies used different measures to define maternal hostility, which could explain discrepancies in findings.

Similarly, findings are inconsistent on the role of positive parenting practices. Among the studies included, one community-based longitudinal study in the United States suggested maternal sensitive parenting practice significantly reduced the link between maternal childhood sexual trauma and child conduct problems (Zvara et al., 2017), whereas two studies conducted in Germany and Canada respectively only found its mediating protective effect between maternal depression and child emotional-behavioural development (Bodeker et al., 2019; Bouvette-Turcot et al., 2020). Furthermore, the UK ALSPAC study found that a mother's higher confidence in her relationship with her child negatively mediated 13% of the total effects of maternal childhood sexual abuse on their children's emotional-behavioural problems (Roberts et al., 2004), while a longitudinal study in Canada found responsive parenting not to significantly mediate the association between MCA and child internalising behaviour (Madigan et al., 2015).

Attachment styles are patterns of interactions in intimate relationships (Widom, Czaja, Kozakowski, & Chauhan, 2018), and thus important family-level factors at theoretical and practical level. Among the three studies investigating the role of maternal–child attachment in the pathway between MCA and children's emotional-behavioural development, two found that maternal avoidant attachment (Pereira et al., 2018) and insecure infant attachment (Pasalich et al., 2016) significantly mediated 35 and 64% of the association between MCA and child externalising problems, while the other study indicated that maternal secure attachment was protective, and mediated 31% of the total effect between MCA and child total behavioural problems (Roth et al., 2021).

Another important family-level factor is intimate partner violence, which was investigated as a potential mediator in four studies (Giallo et al., 2020; Roberts et al., 2017, 2018; Zvara et al., 2017). One of these found that postpartum exposure to intimate partner violence mediated 16% of the total effect between MCA and child emotional-behavioural problems (Giallo et al., 2020), while one study only found it mediated 24% of the total effect between maternal childhood sexual trauma and sensitive parenting (Zvara et al., 2017). However, two population-based case–control studies did not find intimate partner violence to be a mediator between MCA and child ASD or ADHD (Roberts et al., 2017, 2018).

#### Community and Societal- level mediators

Only six studies reported on community factors (social support and psychosocial risks) and no studies on societal factors.

Although social support is generally accepted to be a protective factor for mental health (Wang, Mann, Lloyd-Evans, Ma, & Johnson, 2018), only three studies investigated the mediating role of maternal perceived social support, with one study finding this to be a significant protective mediator (*β* = 0.06, 95% CI 0.01–1.15) in primarily poor African–American mother–child dyads (Min et al., 2013), and the other two studies suggesting its role was non-significant (Bosquet Enlow et al., 2018; Pereira et al., 2018). For infants, a longitudinal study found that the relationship between MCA and infant emotional health was mediated by cumulative psychosocial risks (*β* = 0.037, 95% CI 0.001–0.056), including being a single parent, being a teenage mother, having low family income, low maternal education and marital conflict (Madigan et al., 2017). However, two studies conducted in a large-scale cohort and in a low-income community sample did not find socio-economic circumstances to be significant mediators between MCA and children's ADHD or total behavioural problems, including social withdrawal, anxiety/depression and aggressive behaviour (Roberts et al., 2018; Thompson, 2007).

#### Meta-analysis of single-mediator analyses

**Fig. 4.**
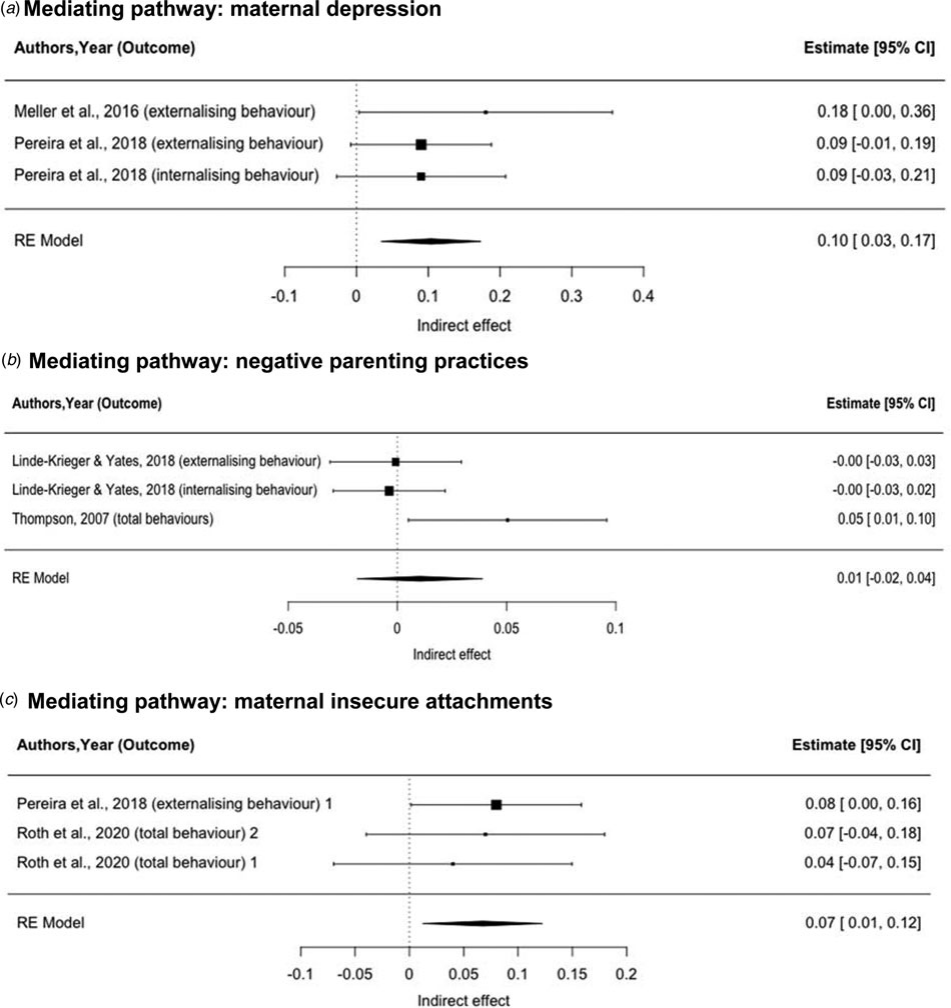
Pooled indirect effects of mediating pathways between maternal childhood adversity and their children's emotional and behavioural problems. (a) Mediating pathway: maternal depression. (b) Mediating pathway: negative parenting practices. (c) Mediating pathway: maternal insecure attachments. *Note*: ^1^ Mediator: avoidant maternal attachment; ^2^ Mediator: anxious maternal attachment.

To limit heterogeneity of findings and ensure reliability, we limited the meta-analysis to studies that assessed emotional and behavioural development in terms of externalising behaviours, internalising behaviours and emotional-behavioural difficulties, assessed using the CBCL and the SDQ. The meta-analysis was therefore conducted on nine single-mediator pathway analyses with complete coefficients (Fig. 3). The standardised indirect effect estimates (*β*s) were 0.10 (95% CI 0.03–0.17) for an individual-level mediating pathway (maternal depression), 0.01 (95% CI −0.02 to 0.04) for a family-level mediating pathway (negative parenting practices) and 0.07 (95% CI 0.01–0.12) for a family-level mediating pathway (maternal insecure attachment) (Fig. 4). Furthermore, 47 and 25% of the total effect of MCA on child behaviours could be mediated by maternal depression and maternal insecure attachments, respectively (online Supplementary Fig. S1).

### Moderators

#### Individual-level moderators

Individual-level moderators included maternal mental health, child sex, biological and genetic factors.

In contrast to its significant mediating role discussed above, two studies reported a non-significant moderating effect of maternal mental health in the association between MCA and children's (Miranda et al., 2011) or infants' emotional and behavioural dysregulation (Bouvette-Turcot et al., 2015). However, a study conducted in the Gaza Strip found that maternal exposure to war trauma acted as a moderator between maternal childhood emotional abuse and infant negative affectivity, with a significant interaction for maternal childhood emotional abuse X war trauma (*β* = −0.21, *p* = 0.002) (Isosavi et al., 2017). A tentative explanation is that high war trauma could blur the maternal perception of their infants' characteristics, as the results of this study were based on maternal ratings of infants' behaviours. Additionally, for adolescent antisocial behaviour, one study found maternal antenatal depression could strengthen the association between MCA and child antisocial behaviours (Plant et al., 2013).

The most frequently investigated moderator in the relationship between MCA and children's emotional-behavioural problems was child sex. Four of the six studies that tested the moderating effect of child sex did not find any sex difference in the association between MCA and child behaviours (Choi et al., 2019; Min et al., 2013; Miranda et al., 2013a; Roberts et al., 2013). However, using moderated mediation and multi-group analyses, the other two studies revealed a sex difference in the mediating pathway of parenting practice between MCA and child externalising behaviour, with the mediating effect of parenting practises being only significant for girls (Linde-Krieger & Yates, 2018; Yoon et al., 2019). These findings suggest that girls may be more sensitive than boys to parenting practices in their emotional and behavioural development.

Other biological and genetic moderators were reported in four studies, including infant serotonin-transporter-linked promoter region (5-HTTLPR), dopamine transporter solute carrier family C6, member 4 (SLC6A3), and catechol- O-methyltransferase (COMT) genes, maternal oxytocin receptor (OXTR, rs53576) genotype and maternal cortisol levels (Bouvette-Turcot et al., 2015; Ludmer et al., 2018; Villani et al., 2018), as well as child frontal alpha wave asymmetry (van de Ven et al., 2020). These moderators were suggested to interact with MCA, further affecting both infant and child emotional-behavioural dysregulation, such as negative emotionality, behavioural dysregulation and mother-infant disorganised attachment.

#### Family-level moderators

Only two studies tested the moderating role of negative parenting practices. A small cross-sectional study found that the mediating effect of maternal postpartum depression was stronger among mothers with high levels of maternal hostility compared to mothers with low levels of maternal hostility (Meller et al., 2016). By contrast, another cross-sectional study in mothers from psychiatric outpatient settings did not find harsh parenting discipline to be a significant moderator in the relationship between MCA and child internalising or externalising behaviours (Miranda et al., 2011).

## Discussion

To the best of our knowledge, this is the first comprehensive systematic review of mediators and moderators in the relationship between maternal childhood adversity and the emotional-behavioural development of their children, and this is also the first meta-analysis of mediating pathways in this link. Our review shows that maternal mental health, child traumatic experiences and insecure maternal–child attachment are consistent mediators. Our meta-analysis also confirms two significant mediating pathways: maternal depression and maternal insecure attachment. However, we found limited evidence on the mediating role of community-level factors, especially societal and cultural factors, pointing to an important research gap in this area. This is further compounded by the fact that the only few studies identified predominantly investigated the moderating role of biological and genetic factors, with no comprehensive evaluation of psychosocial factors.

Maternal depression was confirmed as a key mediator in the relationship between MCA and child emotional-behavioural problems. Notably, multiple studies have found that depression occurring in the first three years postpartum is a significant mediator, highlighting the importance of good maternal perinatal mental health throughout infants' childhood. Interestingly, evidence from low-income communities indicates that mothers with more depressive symptoms might be more likely to use restrictive management strategies to keep children safe from harm (Gutman, Friedel, & Hitt, 2003), suggesting that negative parenting practices, might overtake any potential mediating role for maternal depression. Previous studies also found evidence that the relationship between maternal depression and children's externalising symptoms is partially negatively mediated by the maternal level of positivity in the interaction with her child (Ewell Foster, Garber, & Durlak, 2008). However, the negative impact of experiencing socioeconomic deprivation can also play a significant role in the emotional and behavioural development of the child, masking any specific effect of poor maternal mental health.

A maternal insecure attachment was confirmed as another significant mediator in the association between MCA and child internalising and externalising problems. Adult attachment styles are patterns of interactions in intimate relationships throughout adulthood, developed from expectations and responses to interpersonal events in early childhood relationships (Widom et al., 2018), and are influenced by childhood adversity and family environment. More research would help establish whether other mediators co-occur with maternal attachment in this pathway.

Interestingly, our meta-analysis suggests that negative parenting practices do not significantly mediate the association between MCA and child emotional-behavioural problems (Esteves et al., 2017; Linde-Krieger & Yates, 2018; Thompson, 2007). Importantly, child sex may moderate the mediating role of negative parenting practices, being more evident in girls than in boys (Linde-Krieger & Yates, 2018; Yoon et al., 2019). This points to the potential value of sex-specific interventions if these findings are replicated. Of note, studies that did not find a significant moderating effect of child sex did not evaluate parenting practices as mediators. Girls may be particularly sensitive to parenting styles and negative events occurring in the family context, by virtue of their tendency to have a closer relationship with their families (Smith Leavell & Tamis-LeMonda, 2013). Also, sex differences may be due to different biological responses to childhood stressors, as some evidence suggests that girls have a stronger cortisol response to childhood stressors (Hollanders, van der Voorn, Rotteveel, & Finken, 2017).

By contrast, only few studies investigated positive parenting, pointing to maternal confidence in her relationship with her child as a significant protective mediator (Roberts et al., 2004), while findings on responsive/sensitive parenting were inconsistent (Madigan et al., 2015; Zvara et al., 2017). Of note, converging evidence from both intervention trials and observational longitudinal studies suggests that, at least in early childhood, positive rather than negative parenting may be a developmentally more important predictor of child problem behaviours (Gardner, Hutchings, Bywater, & Whitaker, 2010; Gardner, Shaw, Dishion, Burton, & Supplee, 2007; Gardner, Sonuga-Barke, & Sayal, 1999). Interestingly, a recent study found that responsive parenting acted as a moderator mitigating the ill effects of maternal posttraumatic stress disorder (PTSD) symptoms on children's depression and stress-related symptoms (Greene, McCarthy, Estabrook, Wakschlag, & Briggs-Gowan, 2020). More research would help establish the potential benefit of promoting positive parenting in breaking the intergenerational transmission of childhood adversity on child mental health.

Another important family-level mediating pathway we identified is the exposure of the children themselves to traumatic experiences. This is consistent with evidence that a family may stay locked in an environment of childhood adversity, as supported by meta-analytic evidence that children whose parents have a history of childhood maltreatment are three times more likely to be maltreated than those whose parents have no such history (Assink et al., 2018). Furthermore, research has also suggested that childhood adverse experiences such as maltreatment, often aggregate with low social economic status, family disruption and intimate partner violence, indicating the likely involvement of multiple complex pathways related to the family context (Appleyard et al., 2005).

The presence of intimate partner violence further complicates the rearing environment, with its strong association with maternal mental health, household dysfunction, and parenting negative practices. Here, we found that intimate partner violence mediated the relationship between maternal childhood adversity and child emotional-behavioural problems. Intimate violence severely impacts mothers and child, through maternal exposure to violence and, simultaneously, a traumatic experience for the child (witnessing of violence). Indeed, children who witness intimate partner violence and experience harsh parenting have been found to present more severe behavioural problems (Easterbrooks, Katz, Kotake, Stelmach, & Chaudhuri, 2015). At community level, only few studies investigated the mediating effects of maternal social support and socio-economic status, with inconsistent results. This highlights the need for a more in-depth evaluation of the role of social support, especially among those mothers living in a complex intimate relationship and in socio-economically deprived environments, as they represent a particularly vulnerable population.

Importantly, our finding that some mediators were associated with each other points to the need to evaluate more multilevel-mediator models. For instance, the absence of maternal sensitive parenting could mediate the effect of intimate partner violence on child conduct problems (Zvara et al., 2017). This is in line with previous evidence that having a supportive romantic partner and low levels of intimate partner violence are a buffer against the intergenerational cycle of abuse (Jaffee et al., 2013). In addition, both maternal and paternal hostility and parenting discipline were found to be inter-correlated and both acted as significant mediators between MCA and child externalising behaviours (Rijlaarsdam et al., 2014). This points to the importance of parental roles beyond the maternal one for child mental health, particularly within the family rearing environment.

This review showed that more than 90% of studies identified were conducted in high-income and western countries, whereas most of the world's children and adolescents live in low- and middle-income countries. On one side, this could reflect the fact that we only included studies published in English. However, it could also point to a real need for more studies in low-income countries which face greater financial and human resources constraints in the allocation of their health and social protection resources. In addition, society's attitude and culture can influence family-level factors, another area that demands additional exploration (Dwairy et al., 2010). For example, an effect for maternal parenting practices has been found to be more significant in the Middle East and South Asia, where fathers are less involved in parenting because of cultural differences (Dwairy et al., 2010). Previous evidence has also shown that East Asian mothers may be more psychologically controlling than Western mothers (Pomerantz & Wang, 2009). A cross-national investigation in six countries indicated that a more frequent experience of physical discipline was less strongly associated with adverse child outcomes in countries where the experience of physical discipline is more normative for the cultural context (Lansford et al., 2005). However, whether these cultural differences influence the relationship between MCA and children's emotional-behavioural development remains to be established.

This review has a number of strengths. To our knowledge, this is the first systematic review based on the ecological framework that synthesises the mediators and moderators in the relationship between MCA and children's emotional-behavioural development, and the first to meta-analyse its possible mediating pathways. For example, here we were able to show specificity for the mediating effects of maternal depression and negative parenting practices with a meta-analytic approach, highlighting the role of maternal mental health as a key factor. Importantly, this review points to the urgent need to investigate community and societal -level mediators and moderators, including social support, socio-economic status and cultural differences.

Some limitations should also be addressed. First, we could not include all studies in the meta-analysis due to differences in outcomes and lack of estimates for some of the mediators. Second, all studies were based on retrospective reports of maternal childhood adversity, which might introduce recall and reporting biases, although retrospective reports in adulthood of major adverse experiences in childhood have been validated and they have a worthwhile place in research (Hardt & Rutter, 2004). Third, our review only focused on the traditional maternal role, while many modern families do not follow the traditional nuclear family structure. We focused on mothers as their role has been the most frequently reported in the literature and as they are still the most common carers in many societies, playing a vital role in practical interventions. However, fathers, as well as stepparents, guardians and extended family as caregivers could all play an important role in the pathways to children's outcomes and modern family structures should be more comprehensively evaluated in this context. Lastly, most studies were conducted in high-income western countries as discussed above, which might limit the generalisability of the findings to low- and middle-income countries. Nonetheless, the fact that some studies were conducted in disadvantaged groups, such as teenage mothers, low-income families and clinical settings, would help make the results more generalisable.

## Conclusion

Families, schools, and communities, as well as clinicians could benefit from this evidence for conducting more effective interventions. For example, in primary health settings, physicians should be mindful of the broader implications of maternal depressive symptoms and respond with prompt assessment and further referral for symptom management, particularly in those mothers who also have a personal history of childhood adversity. In maternity and community health settings, an optimal parenting style and higher individual resilience in both mother and child can be promoted to mitigate family-level risk (Avellar & Supplee, 2013). At a societal level, it would be important to increase public awareness on the importance of good mental health and positive parenting. These multilevel interventions informed by established mediators could help minimise the impact of MCA on children's mental wellbeing in the most vulnerable children and their families, helping them pursue a more positive life trajectory.

## Supplementary material

For supplementary material accompanying this paper visit https://doi.org/10.1017/S0033291722001775. click here to view supplementary material
