# Supplementary material for: Mediators and moderators in the relationship between maternal childhood adversity and children's emotional and behavioural development: a systematic review and meta-analysis
Source: Psychol Med. 2022 Jun 22;52(10):1817–37. doi: 10.1017/S0033291722001775 (PMC9340854; doi:10.1017/S0033291722001775)
Supplement: Supplementary file 1 [file S0033291722001775sup.zip › S0033291722001775sup001.docx]

**Appendix A: Search strategy**

The main search strategy was based on the terms listed below and adapted to the requirements of the databases. Subject headings in EMBASE and PsycINFO were used to expand the literature search. To get the maximum number of relevant citations, we used search strings as keywords for studies retrieval. Studies were identified with a search using the combination of **Term 1** AND **Term 2** AND **Term 3**. The search was informed by strategies from earlier studies (Norman et al., 2012(Norman *et al.*, 2012); Lange et al., 2019(Lange *et al.*, 2019); Fritz et al., 2018(Fritz *et al.*, 2018); Langevin et al., 2019(Langevin *et al.*, 2019); Meng et al., 2018(Meng *et al.*, 2018); Ximenes et al., 2019(Ximenes *et al.*, 2019)).

**Term 1:** (maternal child* OR maternal history OR mother* history).tw. AND ((advers* OR trauma* OR traged* OR abus* OR illtreat* OR ill-treat* OR mal-treat* OR maltreat* OR mistreat* OR ACE* OR assault* OR violen* OR neglect* OR victimization OR aggression OR conflict OR offense* OR antipathy).tw. OR exp *child abuse/ or exp *child neglect/ or exp *emotional abuse/ or exp *psychological abuse/ or exp *verbal abuse/ or exp *physical abuse/ or exp *sexual abuse/ or exp * emotional neglect/ or exp *trauma/ or exp *victimization/ or exp *childhood adversity/ or exp *childhood trauma/ or exp *antipathy/)

**Term 2:** (resilien* OR protect* OR support* OR adapt* OR promot* OR moderat* OR mediat* OR predict* OR associate* OR influenc* OR risk* OR outcome* OR consequence* OR impact* OR pathway* OR nurturing* OR psychosocial).tw. OR exp "Resilience (Psychological)"/ OR exp *protective factors/ OR exp *risk factors/ OR exp *adaptability/ OR exp *adjustment/ OR exp *coping behaviour/ OR exp *adaptive behaviour/ OR exp *parenting style/ OR exp *parental role/ OR exp *parental involvement/ OR exp *parental expectations/ OR exp *sibling relations/ OR exp *intergenerational relations/ OR exp *transgenerational patterns/ OR exp *generational differences/ OR exp *home environment/

**Term 3:** (bab* OR newborn* OR neonate* OR infant* OR child* OR adolescen* OR teen* OR offspring* OR intergeneration* OR transgeneration* OR generation*).tw. AND ("self-harm*" OR suicid* OR psychopatholog* OR psycholog* OR psychiatr* OR emotion* OR affect* OR mental* OR disorder* OR development* OR behavio* OR problem* OR dysfunction OR distress OR depress* OR anxi* OR psychos* OR conduct OR antisoci* OR disruptive OR internal OR external OR adjustment OR maladjustment OR attention OR hyperactivit* ).tw. OR exp *offspring/ OR exp *mental health/ OR exp *dysfunction/ OR exp *distress/ OR exp *stress/ OR exp *mental illness/ OR exp *mental development/ OR exp *psychopathology/ OR exp *conduct/ OR exp *intergenerational OR exp *maladjustment/ OR exp *adjustment/ OR exp *attention/ OR exp *hyperactivity/

**References**

**Fritz, J., de Graaff, A. M., Caisley, H., van Harmelen, A. L. & Wilkinson, P. O.** (2018). A Systematic Review of Amenable Resilience Factors That Moderate and/or Mediate the Relationship Between Childhood Adversity and Mental Health in Young People. *Frontiers in psychiatry* **9**230. doi:<https://doi.org/10.3389/fpsyt.2018.00230>

**Lange, B. C. L., Condon, E. M. & Gardner, F.** (2019). A systematic review of the association between the childhood sexual abuse experiences of mothers and the abuse status of their children: Protection strategies, intergenerational transmission, and reactions to the abuse of their children. *Social Science & Medicine* **233**113-137. doi:<https://doi.org/10.1016/j.socscimed.2019.05.004>

**Langevin, R. A.-O. X., Marshall, C. & Kingsland, E.** (2019). Intergenerational Cycles of Maltreatment: A Scoping Review of Psychosocial Risk and Protective Factors. *Trauma, Violence, & Abuse* **22**(4), 672–688. doi:<https://doi.org/10.1177/1524838019870917>

**Meng, X. A.-O., Fleury, M. J., Xiang, Y. T., Li, M. & D'Arcy, C.** (2018). Resilience and protective factors among people with a history of child maltreatment: a systematic review. *Social psychiatry and psychiatric epidemiology* **53**(5), 453-475. doi:<https://doi.org/10.1007/s00127-018-1485-2>

**Norman, R. E., Byambaa M Fau - De, R., De R Fau - Butchart, A., Butchart A Fau - Scott, J., Scott J Fau - Vos, T. & Vos, T.** (2012). The long-term health consequences of child physical abuse, emotional abuse, and neglect: a systematic review and meta-analysis. *PLoS Medicine* **9**(11), e1001349. doi:<https://doi.org/10.1371/journal.pmed.1001349>

**Ximenes, R. B. B., Ximenes, J. C. M., Nascimento, S. L., Roddy, S. M. & Leite Á, J. M.** (2019). Relationship between maternal adverse childhood experiences and infant development: A systematic review (protocol). *Medicine* **98**(10), e14644. doi:<https://doi.org/10.1097/MD.0000000000014644>
