# Supplementary material for: Mediators and moderators in the relationship between maternal childhood adversity and children's emotional and behavioural development: a systematic review and meta-analysis
Source: Psychol Med. 2022 Jun 22;52(10):1817–37. doi: 10.1017/S0033291722001775 (PMC9340854; doi:10.1017/S0033291722001775)

**Appendix B: Supplementary files**

**Table S1.** **Quality assessment of the cross-sectional studies included according to Newcastle-Ottawa Scale**

| **Study** | **Design** | **Selection** | | | | **Comparability** | **Outcome** | | **Total score** |
| --- | --- | --- | --- | --- | --- | --- | --- | --- | --- |
|  |  | **Representative sample** | **Adequate sample size** | **Non-respondents** | **Ascertainment of exposure** | **Based on design or analysis** | **Assessment of outcome** | **Statistical test** |  |
| Warmingham et al., 2020 | Cross-sectional | - | - | + | - | ++ | ++ | + | 6 |
| Ludmer et al., 2018 | Cross-sectional | - | - | - | - | ++ | ++ | + | 5 |
| Bodeker et al., 2019 | Cross-sectional | - | - | - | + | ++ | + | + | 5 |
| Meller et al., 2016 | Cross-sectional | + | - | - | - | ++ | + | + | 5 |
| Miranda et al., 2013 (a) | Cross-sectional | - | - | + | - | ++ | ++ | + | 6 |
| Miranda et al., 2013 (b) | Cross-sectional | - | - | + | - | ++ | ++ | + | 6 |
| Esteves et al., 2017 | Cross-sectional | + | - | - | - | ++ | + | + | 5 |
| Villani et al., 2018 | Cross-sectional | + | - | - | - | ++ | ++ | + | 6 |
| Miranda et al., 2011 | Cross-sectional | + | - | + | - | ++ | + | + | 6 |
| Oshio & Umeda, 2016 | Cross-sectional | + | - | - | - | ++ | + | + | 5 |
| Russotti et al., 2021 | Cross-sectional | + | - | + | - | + | + | + | 5 |

**Table S2.** **Quality assessment of the case-control studies included according to Newcastle-Ottawa Scale**

| **Study** | **Design** | **Selection** | | | | **Comparability** | **Exposure** | | | **Total score** |
| --- | --- | --- | --- | --- | --- | --- | --- | --- | --- | --- |
|  |  | **Adequate Case Definition** | **Case representative** | **Selection of controls** | **Definition of Controls** | **Based on design or analysis** | **Ascertainment of exposure** | **Same method for cases and controls** | **Non-response rate** |  |
| Roberts et al., 2013 | Case control | + | + | + | + | ++ | - | + | + | 8 |
| Roberts et al., 2017 | Case control | + | + | + | + | ++ | - | + | - | 7 |
| Roberts et al., 2018 | Case control | + | + | + | + | ++ | - | + | - | 7 |

**Table S3. Quality assessment of the cohort studies included according to Newcastle-Ottawa Scale**

| **Study** | **Design** | **Selection** | | | | **Comparability** | **Outcome** | | | **Total score** |
| --- | --- | --- | --- | --- | --- | --- | --- | --- | --- | --- |
|  |  | **Representative exposed cohort** | **Selection of non-exposed cohort** | **Ascertainment of exposure** | **Outcome not present at start of study** | **Based on design or analysis** | **Assessment of outcome** | **Follow-up time** | **Adequate follow-up** |  |
| Giallo et al., 2020 | Cohort | + | + | - | + | ++ | - | + | + | 6 |
| Bouvette-Turcot et al., 2015 | Cohort | - | + | - | + | + | - | + | + | 5 |
| Collishaw et al., 2007 | Cohort | + | + | - | + | + | - | + | + | 6 |
| Bosquet Enlow et al., 2018 | Cohort | + | + | + | + | ++ | - | + | + | 8 |
| Linde-Krieger & Yates, 2018 | Cohort | + | + | + | + | ++ | + | + | + | 9 |
| Pereira et al., 2018 | Cohort | - | + | - | + | ++ | - | + | + | 6 |
| Choi et al., 2019 | Cohort | + | + | + | + | ++ | - | + | + | 8 |
| Madigan et al., 2017 | Cohort | + | + | + | + | ++ | - | + | + | 8 |
| Liu et al., 2019 | Cohort | - | + | - | + | ++ | + | + | + | 7 |
| Yoon et al., 2019 | Cohort | + | + | - | + | ++ | - | + | + | 7 |
| Isosavi et al., 2017 | Cohort | + | + | - | + | ++ | - | + | + | 7 |
| Plant et al., 2017 | Cohort | + | + | - | + | ++ | - | + | + | 7 |
| Choi et al., 2017 | Cohort | + | + | - | + | ++ | - | + | - | 6 |
| Pasalich et al., 2016 | Cohort | + | + | + | + | ++ | - | + | + | 8 |
| Madigan et al., 2015 | Cohort | + | + | - | + | ++ | - | + | - | 6 |
| Myhre et al., 2014 | Cohort | + | + | - | + | ++ | - | + | + | 7 |
| Min et al., 2012 | Cohort | - | + | - | + | ++ | - | + | + | 6 |
| Plant et al., 2013 | Cohort | + | + | - | + | ++ | - | + | + | 7 |
| Thompson, 2007 | Cohort | + | + | - | + | ++ | - | + | + | 7 |
| McDonnell & Valentino, 2016 | Cohort | + | + | - | + | ++ | - | + | + | 7 |
| Zvara et al., 2017 | Cohort | + | + | - | + | ++ | - | + | + | 7 |
| Rijlaarsdam et al., 2014 | Cohort | + | + | - | + | ++ | - | + | + | 7 |
| Roberts et al., 2004 | Cohort | + | + | - | + | ++ | - | + | + | 7 |
| Bouvette-Turcot et al., 2020 | Cohort | + | + | - | + | ++ | - | + | + | 7 |
| Roth et al., 2020 | Cohort | - | + | - | + | ++ | - | + | + | 6 |
| van de Ven et al., 2020 | Cohort | + | + | - | + | ++ | - | + | + | 7 |
| Linde-Krieger &Yates, 2021 | Cohort | + | + | + | + | ++ | - | + | + | 8 |

**Table S4. Studies excluded at full-text screening**

| **Study** | **Excluding reason** |
| --- | --- |
| Powers A. et al, 2020 | Not in line with maternal exposure criteria |
| Claridge, A. M. et al, 2014 | Does not separate maternal childhood and adulthood adversity |
| Bouvette-Turcot, AA. et al, 2013 | Not in line with maternal exposure criteria |
| Morrel, T. M. et al, 2003 | Does not separate maternal childhood and adulthood adversity |
| Fuchs A. et al, 2017 | Not in line with child outcome criteria |
| Toepfer P. et al, 2019 | Not in line with child outcome criteria |
| Condon E.M. et al, 2019 | No mediating/moderating analysis of the relationship between MCA and child outcomes |
| Donald K.A. et al, 2019 | Not in line with child outcome criteria |
| Swartz H.A. et al, 2018 | Not in line with our research question |
| Kluczniok D. et al, 2016 | Not in line with our research question |
| Baril K. et al, 2016 | No mediating/moderating analysis in the relationship between MCA and child outcomes |
| Bifulco A. et al, 2002 | MCA not the main factor under investigation and analysis |
| Khan, M. & Renk, K., 2018 | Not in line with child outcome criteria |
| Yehuda, R. & Meaney, M. J., 2018 | Not in line with child outcome criteria |
| Henschel, S. et al, 2018 | Not in line with our research question |
| Hipwell, AE. et al, 2019 | Not in line with our research question |
| Lang A.J.et al, 2010 | No mediating/moderating analysis in the relationship between MCA and child outcomes |
| Lyons-Ruth K. et al, 2003 | No mediating/moderating analysis in the relationship between MCA and child outcomes |
| Hatzis D. et al, 2019 | Not in line with child outcome criteria |
| Dittrich K. et al, 2018 | Not in line with child outcome criteria |
| Riva Crugnola C. et al, 2019 | Not in line with child outcome criteria |
| Gartland D. et al, 2019 | No mediating/moderating analysis in the relationship between MCA and child outcomes |
| Roberts A.L., et al, 2015 | Does not separate maternal childhood and adulthood adversity |
| Wu Q. & Slesnick N., 2020 | Not an observational study |
| Kankaanpaa, S. L, et ak, 2020 | Not in line with child outcome criteria |
| Galbally M. et al, 2020 | No mediating/moderating analysis in the relationship between MCA and child outcomes |
| Chang Y.-T. et al, 2020 | Not in line with child outcome criteria |
| Wadji D.L. et al, 2020 | No mediating/moderating analysis in the relationship between MCA and child outcomes |
| Alto M.E. et al, 2020 | Not an observational study |
| Pilkay S.R. et al, 2020 | Not in line with child outcome criteria |
| Koverola C et al, 2005 | Does not separate maternal childhood and adulthood adversity. |
| Tanja Hillberg, 2010 | Not an observational study |
| Dubowitz H et al, 2001 | Does not separate maternal childhood and adulthood adversity. |

Note: MCA: maternal childhood adversity.

**Figure S1. Pooled total effects between maternal childhood adversity and their children’s emotional and behavioural problems.**


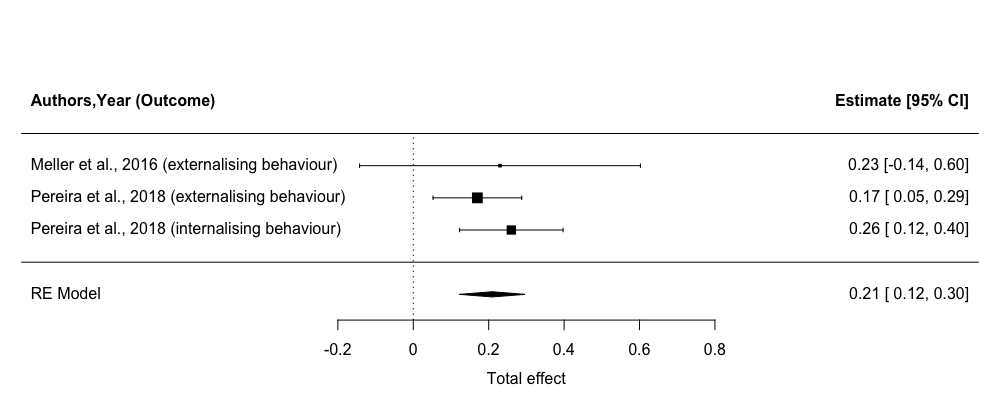


**a. Mediating pathway: maternal depression**

**b. Mediating pathway: negative parenting practices**

**c. Mediating pathway: maternal insecure attachments**

**Note: ^1^ Mediator: avoidant maternal attachment; ^2^ Mediator: anxious maternal attachment.**


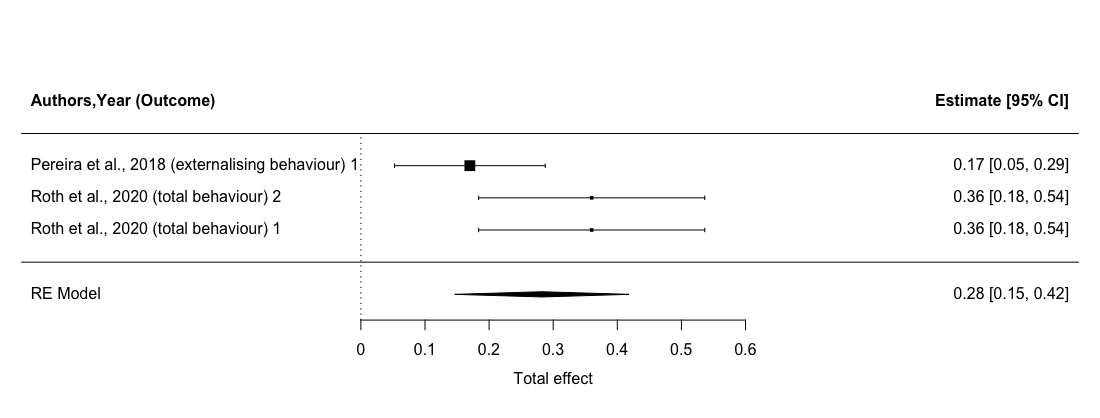

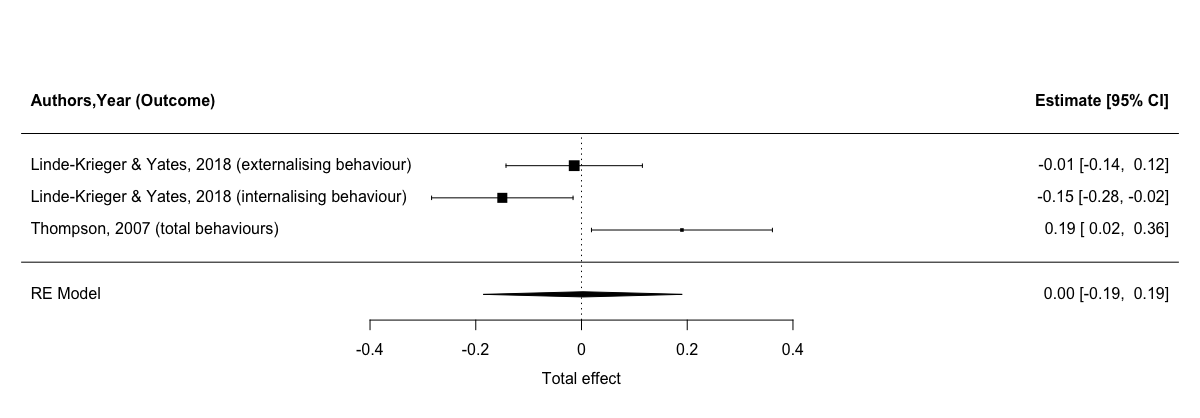

Supplement: Supplementary file 1 [file S0033291722001775sup.zip › S0033291722001775sup002.docx]
